# SEC-Translocon Dependent Extracytoplasmic Proteins of *Candidatus Liberibacter* asiaticus

**DOI:** 10.3389/fmicb.2016.01989

**Published:** 2016-12-20

**Authors:** Samiksha Prasad, Jin Xu, Yunzeng Zhang, Nian Wang

**Affiliations:** Citrus Research and Education Center, Department of Microbiology and Cell Science, Institute of Food and Agricultural Sciences, University of Florida, Lake AlfredFL, USA

**Keywords:** citrus HLB, Liberibacter, Virulence Factors, Sec pathway, secretion

## Abstract

Citrus Huanglongbing (HLB) is the most destructive citrus disease worldwide. HLB is associated with three species of the phloem-limited, gram-negative, fastidious α-proteobacteria: *Candidatus* Liberibacter asiaticus (Las), *Ca.* L. americanus (Lam), and *Ca.* L. africanus (Laf) with Las being the most widespread species. Las has not been cultured in artificial media, which has greatly hampered our efforts to understand its virulence mechanisms. Las contains a complete Sec-translocon, which has been suggested to transport Las proteins including virulence factors into the extracytoplasmic milieu. In this study, we characterized the Sec-translocon dependent, signal peptide containing extracytoplasmic proteins of Las. A total of 166 proteins of Las-psy62 strain were predicted to contain signal peptides targeting them out of the cell cytoplasm via the Sec-translocon using LipoP, SigalP 3.0, SignalP 4.1, and Phobius. We also predicated SP containing extracytoplasmic proteins for Las-gxpsy and Las-Ishi-1, Lam, Laf, *Ca.* L. solanacearum (Lso), and *L. crescens* (Lcr). For experimental validation of the predicted extracytoplasmic proteins, *Escherichia coli* based alkaline phosphatase (PhoA) gene fusion assays were conducted. A total of 86 out of the 166 predicted Las proteins were experimentally validated to contain signal peptides. Additionally, Las-psy62 *lepB* (CLIBASIA_04190), the gene encodes signal peptidase I, was able to partially complement the amber mutant of *lepB* of *E. coli*. This work will contribute to the identification of Sec-translocon dependent effector proteins of Las, which might be involved in virulence of Las.

## Introduction

Citrus Huanglongbing (HLB) is the most destructive disease for citrus industry worldwide. HLB is associated with three species of the phloem-limited, gram-negative, fastidious α-proteobacteria: *Candidatus* Liberibacter asiaticus (Las), *Ca.* L. americanus (Lam), and *Ca.* L. africanus (Laf; Capoor et al., 1967; Jagoueix et al., 1996; Bové, 2006). Liberibacters are vectored by two psyllid species, *Diaphorina citri* Kuwayama (ACP) or *Trioza erytreae* (Del Guercio; Halbert and Manjunath, 2004). Las is the widest spread and most virulent species and so far is the only one reported in the US (Gottwald et al., 2010; Wang and Trivedi, 2013).

Besides HLB, Liberibacters are also known to cause many other plant diseases (Jagoueix et al., 1994; Teixeira et al., 2005; Hansen et al., 2008; Liefting et al., 2008; Raddadi et al., 2011) For example, *Ca.* L. solanacearum (Lso; Liefting et al., 2009) has been known to cause Zebra chip of potato and to infect peppers and tomatoes. On the other hand, *Ca*. L. europaeus has been suggested as an endophyte rather than a pathogen (Raddadi et al., 2011). With except *L. crescens* (Lcr), which was originally isolated from mountain papayas (Leonard et al., 2012; Fagen et al., 2014a), most other Liberibacters have not been cultured in artificial media, therefore, traditional molecular and genetic analyses are difficult to apply. This has greatly hampered our efforts to understand the virulence mechanisms of Las. So far, most insights of the HLB biology and Las pathogenicity are derived from the genome sequences of Las and other related Liberibacters including Las, Lam, Laf, *Ca*. Lso, and Lcr (Duan et al., 2009; Lin et al., 2011; Leonard et al., 2012; Fagen et al., 2014b; Wulff et al., 2014).

One of the most important virulence factors of bacterial pathogens is the presence of protein secretion systems, which secrete proteins, called effectors, into host cells. Interestingly, Las contains a complete General Secretory Pathway (GSP/Sec-translocon), but lacks the Sec-dependent type II (T2SS) and type V (T5SS) secretion systems and type III (T3SS) secretion system (Duan et al., 2009). The Sec machinery facilitates the majority of protein transport across the cytoplasmic membrane and is essential for bacterial viability (Segers and Anné, 2011). The Sec pathway is also critical for secretion of important virulence factors by certain bacterial pathogens, e.g., Phytoplasma, a bacterial pathogen residing in the phloem similarly as Las.

Bacterial proteins translocated exclusively by the Sec-translocon are synthesized initially as protein precursors in the cytoplasm, containing signal peptide (SP) sequences of approximately 20–30 amino acid residues at the amino-terminal (Economou, 1999). Proteins containing these SP have a similar architecture and are normally cleaved by signal peptidases: (i) a basic “n region” at the amino terminus, which is about 5–8 amino acids long and is characterized by the presence of basic residues. The net positive charge of this region is known to be crucial for interaction with the negatively charged surface of the inner membrane (Rehm et al., 2001; Palmer and Berks, 2012). (ii) a hydrophobic “h region” in the middle, about 8–12 amino acids long. It is composed largely of non-polar amino acids. This region has a high propensity for alpha-helical formation, a conformation that may facilitate interaction with the interior of the bilayer (Berks, 1996; Palmer and Berks, 2012) and (iii) a polar “c region” or cleavage region about 6 amino acids long at the carboxyl terminus. This region is involved in signal peptidase recognition and cleavage, which is usually required to achieve final folding and localization of the exported proteins (Tuteja, 2005; Palmer and Berks, 2012). The characteristic tripartite amino acid composition in the SP sequences of Sec-translocon dependent pre-proteins is particularly useful to distinguish proteins containing SP (Pugsley, 1993). Numerous dedicated bioinformatics tools are available for predicting the potential localization and eventual destination of the proteins based on the protein sequence (Andersson and von Heijne, 1994).

We hypothesized that Sec-translocon serves as a potent system for the transportation of Las proteins into the extracytoplasmic milieu, which can be identified by the presence of signal peptide sequence. We comprehensively identified Sec-dependent cytoplasmic proteins containing SP in Las and other sequenced Liberibacters using four well-adopted algorithms, and validated the bioinformatic predictions for SP-containing Sec-dependent cytoplasmic proteins in Las using the *Escherichia coli*-based PhoA assay.

## Materials and Methods

### Prediction of Sec-Dependent Extracytoplasmic Proteins of Liberibacters

The entire annotated genome of Las strain Psy62 (taxid: 537021, GenBank accession no. CP001677; Duan et al., 2009). Las strain gxpsy (taxid: 1174529, GenBank accession no. CP004005; Lin et al., 2013); Las strain ishi-1 (taxid: 931202, GenBank accession no. NZ_AP014595; Katoh et al., 2014); Lam strain São Paulo (taxid: 1261131, GenBank accession no. CP006604; Wulff et al., 2014); Laf strain PTSAPSY (taxid: 1277257, GenBank accession no. CP004021; Lin et al., 2015); Lso strain ZC1 (taxid: 658172, GenBank accession no. CP002371; 16) and Lcc strain BT-1 (taxid: 1215343, GenBank accession no. CP003789; Leonard et al., 2012) were screened to identify the genes encoding proteins containing SP. The SP prediction was conducted using the following online algorithms: LipoP server 1.0 (Juncker et al., 2003), Phobius (Käll et al., 2004, 2007), SignalP version 3.0 (Bendtsen et al., 2004), and SignalP version 4.1 (Petersen et al., 2011). The screening was performed with default settings of the algorithms for gram-negative bacteria.

### Ortholog Cluster Homology Analysis of SP Containing Proteins

Genome-wise orthologous gene clustering among the seven strains were performed using Get_homologs program (ver. 20140311) with parameters: -M, -e 0, -E 0.01 and -S 60 (Contreras-Moreira and Vinuesa, 2013). The ANIm values between genomes were calculated using the NUCmer algorithm v3.1integrated in Jspecies v1.2.1 (Richter and Rossello-Mora, 2009). The orthologous relationship of the identified SP positive genes were determined based on the orthologous gene clusters generated by Get_homologs. Manual curation was performed for the genes whose original annotation was not proper. A total of 596 clusters of orthologs were generated in this analysis across the seven genomes. The hierarchical clustering of the seven Liberibacters was conducted based on gene presence and absence matrix of the orthologous clusters. Dendro UPGMA^1^ was used to generate the UPGMA tree with Jaccard coefficient. A total of 100 bootstrap replicates were prepared, and the values of >50% at each node was noted as a percent value.

### Gene Specific Primer Design

Gene specific forward and reverse primers for each of the 166 predicted SP containing extracytoplasmic proteins of Las-psy62 strain were designed for amplification of the full-length gene (excluding the stop codon; Supplementary Table S9). The melting temperature and GC content of the primers were calculated^2^. The primers were designed to incorporate appropriate restriction enzyme sites at the 5′ and 3′ ends of the resultant amplicons (Supplementary Table S9).

### Las Genomic DNA Extraction

Huanglongbing symptomatic leaves from citrus groves of Citrus Research and Education Center (CREC), University of Florida, Lake Alfred, Florida were collected and washed with sterilized double distilled water and the midrib section of the leaf was used for extraction of Las genomic DNA. DNA was extracted using the Wizard Genomic DNA purification kit (Promega).

### Alkaline Phosphatase (PhoA) Assays

Gene specific forward and reverse primers were used for amplification of the Las genes. The resultant amplified PCR products were digested with the cognate restriction enzymes (NEB) and subsequently purified by Wizard SV Gel and PCR Clean-Up System (Promega). The fragments were then subjected to ligation with pJDT1-SDM-1 vector using T4 DNA ligase (NEB) to obtain an in-frame gene fusion with *phoA.* The amplified Las genes do not contain the stop codon, and the *phoA* is truncated without its SP sequence for in-frame fusion purpose. The *E. coli* chemically competent strain of JM105 (Promega) was used for transformation.

The transformants were selected on LB agar plates containing 100 μg/mL Ampicillin. The transformants were tested for PhoA activity on LB agar plates containing 90 μg/mL 5- BCIP as chromogenic substrate. To block endogenous phosphatase activity, 75 mM Na_2_HPO_4_ was added. SP presence was indicated by blue colonies, whereas lack of PhoA activity was signified by the white colonies. The plasmids from PhoA positive colonies were purified and sequenced with primers adjacent to the location of insertion (5′-CAG GAA ACA GCT ATG AC-3′; 5′-CGC TAA GAG AAT CAC GCA GAG C-3′ as forward and reverse primers, respectively) for confirmation. The empty pJTD1-SDM-1 vector transformed JM105 competent cells were used as a negative control.

### Multiple Sequence Alignment

The DNA sequences of the *lepB* gene encoding the SPase I in *E. coli* and Las strains were retrieved from National Center of Biotechnology Information (NCBI). Multiple sequence alignment was conducted using Multiple Sequence Comparison by Log Expectation (MUSCLE) with default settings. For the phylogenetic tree and identity matrix of the sequences, the ClustalO (Clustal Omega) version 2.1 at default settings was used.

### Screening for Complementation with Las *lep* Gene

The *E. coli* K-12 MG1655 wild type (IT42: lep) and amber mutant (IT41: *lep9*, or Δ*lep*) strains were grown from stocks received from Dr. Inada at Kyoto University, Japan on LB plates at 37°C overnight with 20 μg/mL tetracycline as selection marker. Single colonies were picked for further studies. The Las *lepB* gene was amplified, flanked by appropriate restriction sites (*Hin*dIII and *Spe*I) for insertion into the pBBR1mcs5 vector. The amplified fragment was digested with appropriate restriction enzymes (NEB) and purified with Wizard^®^ SV Gel and PCR Clean-Up System (Promega). The construct was subjected to ligation with the pBBR1mcs5 vector with T4 DNA ligase (NEB). The chemically competent strain of *E. coli* JM105 (Promega) was used for transformation. The resultant plasmid transformed into the amber mutant of *E. coli* (*lep^-^)* by electroporation. The three strains: *E. coli* wild type (WT), *E. coli* amber mutant (Δ*lep*) and *E. coli* amber mutant complimented with Las_psy62 *lep* (Δ*lep::lep_Las_)* were grown at 32 and 42°C to assess the bacterial growth.

## Results

### Prediction of SP Containing Proteins for Liberibacters

**FIGURE 1:**
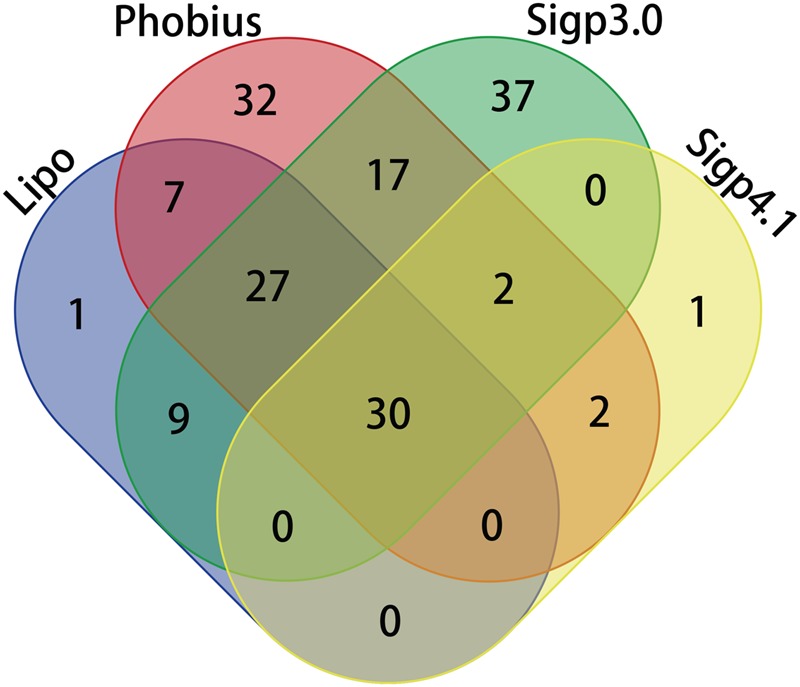
**Venn diagram comparing prediction of Sec-translocon dependent extracytoplasmic proteins of Las using different algorithms**.

A total of 166 proteins were predicted to contain signal peptides in Las-psy62, comprising 15% of the total annotated proteins of Las using LipoP, SigalP 3.0, Signal P4.1, and Phobius (**Tables 1** and **2**). The four tools use distinct algorithms for signal peptide prediction and complement each other, thus the merged list from the four tools comprehensively represented the potential signal peptide containing proteins in Las-psy62 (**Figure 1**). LipoP server 1.0 also categorized proteins into lipoprotein and non-lipoprotein.

**Table 1:** Prediction of signal peptide containing extracytoplasmic proteins in Las-psy62 and PhoA assay results.

Signal Peptide Analysis	Signal peptide prediction by the following algorithms				
	Signal P 4.1	Signal P 3.0 (HMM)	Signal P 3.0 (NN)	Lipo P 1.0	Phobius
Proteins with predicted signal peptide	35	98	65	74	117
Signal peptides tested positive with the PhoA assay	31	63	47	59	67
Predicted SP positive for PhoA assay	89%	64%	72%	80%	57%

**Table 2:** Prediction of signal peptide containing extracytoplasmic protein predictions in different species and strains of Liberibacter.

Proteins with predicted signal peptide	Signal peptide prediction using different algorithms					
	SignalP 4.1	SignalP 3.0(HMM)	SignalP 3.0 (NN)	LipoP 1.0	Phobius	Total number of predictions
Las-psy62	35	98	65	74	117	166
Las-gxpsy	37	100	68	75	118	168
Las-ishi-1	34	97	68	71	116	164
Lam	25	86	55	56	81	133
Laf	31	77	64	53	89	141
Lso	49	106	77	79	120	171
Lcc	42	149	106	103	149	214

Prediction of SP containing proteins was performed for six more *Liberibacter* strains including two more Las strains, another two species also causing HLB (Lam and Laf), one non-citrus pathogenic species Lso and one non-pathogenic relative species Lcc (**Table 2**, Supplementary Tables S1–S6). Las-gxpsy and Las-ishi-1 were predicted to have a total of 168 (Supplementary Table S1) and 164 (Supplementary Table S2) SP containing extracytoplasmic proteins, respectively. Lam contains 133 (Supplementary Table S3) predicted SP containing proteins, whereas Laf contains 141 (Supplementary Table S4). For Lso 171 SP containing proteins were predicted (Supplementary Table S5). A total of 214 putative SP containing proteins were predicated for Lcc (Supplementary Table S6).

### Orthologous Cluster Homology Analysis of SP Containing Proteins

**FIGURE 2:**
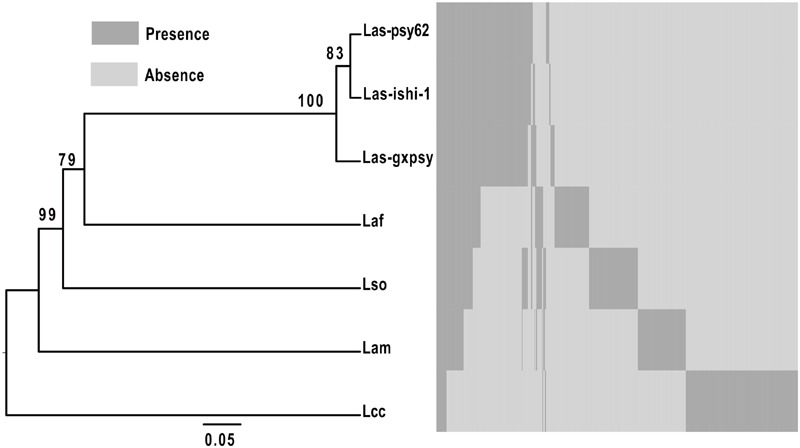
**Hierarchical clustering of putative extracytoplasmic proteins of seven Liberibacter species and strains based on orthologous clusters.** The hierarchical clustering of the seven Liberibacters was conducted based on gene presence and absence matrix of the orthologus clusters. Dendro UPGMA (http://genomes.urv.es/UPGMA/) was used to generate the UPGMA tree with Jaccard coefficient. A total of 100 bootstrap replicates were prepared, and the values of >50% at each node was noted as a percent value. Las-psy62 = *Ca*. L. asiaticus strain psy62, Las-gxpsy = *Ca*. L. asiaticus strain gxpsy, Las-ishi-1 = *Ca*. L. asiaticus strain ishi-1, Laf = *Ca*. L. africanus strain PSTAPSY, Lam = *Ca*. L. americanus strain Sao Paulo, Lso = *Ca*. L. solanacearum strain CZ1 and Lcc = *L. crescens* strain BT1.

Orthologous relationship between the identified putative extracytoplasmic proteins of the seven sequenced Liberibacters was determined (**Figure 2**, Supplementary Table S7). 596 orthologous clusters were formed when the threshold identity 60% and coverage 75% was applied. This analysis allowed us to compare the predicted SP containing extracytoplasmic proteins of different strains and species of *Liberibacter*. Interestingly, this phyletic tree based on the distribution of the SP positive proteins among the seven strains is consistent with the maximum-likelihood phylogenetic trees reconstructed using 16S rRNA gene sequences (Fagen et al., 2014a), indicating the gain and loss history of these SP positive proteins was convergent with the evolution history of the relevant genome background.

Only 17 predicted extracytoplasmic proteins are homologous between the seven Liberibacters (**Table 3**, Supplementary Table S7). Amongst the six infectious Liberibacters, i.e., Las, Lam, Laf, and Lso, 45 SP containing proteins were predicted. Totally 151 SP containing proteins were shared among the three strains of Las (Supplementary Table S8). 73, 60 and 45 SP containing homologous proteins were shared by Laf, Lso, and Lam, respectively, to Las.

**Table 3:** Common homologs between the seven different species and strains of Liberibacter^∗^.

ID	Annotation
CLIBASIA_00265^∗^	Cationic amino acid ABC transporter, periplasmic binding protein
CLIBASIA_00400	CTP synthetase
CLIBASIA_00560	Pyrophosphate–fructose-6-phosphate 1 -phosphotransferase
CLIBASIA 01020	Large subunit ribosomal protein L35
CLIBASIA_01295^∗^	Flagellar basal body L-ring protein
CLIBASIA_01305^∗^	Flagellar basal body P-ring protein
CLIBASIA_01315^∗^	Flagellar basal body rod protein FlgG
CLIBASIA 01765	Sensory box/GGDEF family protein
CLIBASIA_02160^∗^	Metalloprotease
CLIBASIA 02865	Flagellar motor protein MotA
CLIBASIA_03145	Hypothetical protein
CLIBASIA 03450	DNA translocase FtsK
CLIBASIA_03680^∗^	Phosphatidylcholine synthase protein
CLIBASIA 04205	UDP-*N*-acetylglucosamine pyrophosphorylase protein
CLIBASIA 04290^∗^	Putative hydrolase serine protease
CLIBASIA_04750^∗^	Malate dehydrogenase
CLIBASIA 05000	Cell division protein FtsW

*^∗^Proteins which are PhoA positive*

### Using *E.coli* as a Model to Indirectly Validate the Predicated SP Containing Proteins with PhoA Assay

**FIGURE 3:**
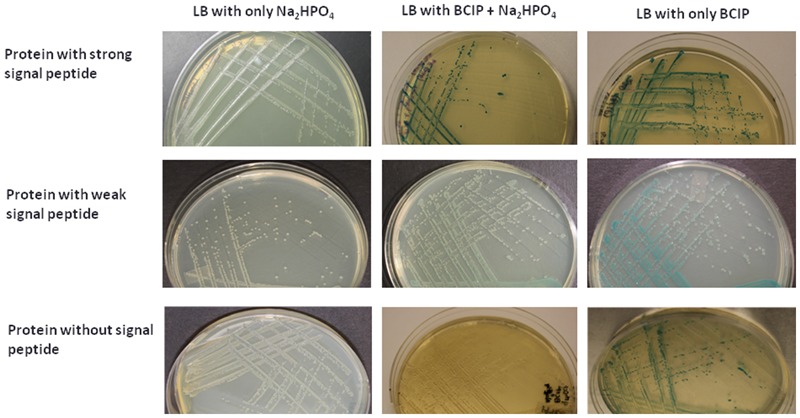
**PhoA assay confirmed the secretion of 86 Las Sec-translocon dependent extracytoplasmic proteins.** *E. coli* expressing candidate-PhoA fusion proteins were grown on LB medium containing BCIP. Representative fusion proteins with strong **(Upper)**, weak **(Middle)**, and no **(Lower)** secretion are presented. 75 mM Na2HPO4 was added to the medium in order to suppress the endogenous phosphatase activity.

To experimentally validate the presence of SP in the predicted SP containing proteins in Las-psy62 strain, PhoA assay was conducted using *E.coli* as a model since SP is highly conserved among different bacteria (Ammerman et al., 2008). Gene specific primer sets for each gene encoding the predicted proteins were designed (Supplementary Table S9). The amplified DNA sequence encoding the putative SP containing protein was inserted upstream of the *phoA* without SP in frame. Out of the 166 predicted proteins, 86 proteins (52%; **Table 1** and Supplementary Table S10) were PhoA positive and turned dark blue at the presence of bromo-4-chloro-3-indolyl phosphate (BCIP; **Figure 3**), suggesting that they contain a SP in their sequences that can direct them to translocate outside of the cytoplasm via the Sec pathway. The empty PJDT1-SDM-1 was used as a negative control, which did not result in color changes. Fifty one predicted proteins were PhoA negative whereas 29 predicted proteins could not be determined experimentally (Supplementary Table S10).

### SPase I Is Conserved in *E. coli* and Las Strains

Type I signal peptidase (SPase I) is responsible for cleaving off the amino-terminal signal peptide from proteins that are secreted across the bacterial cytoplasmic membrane (Paetzel, 2014). We further test whether SPase I is conserved in Las and *E.coli*. Multiple sequence alignment was conducted for SPase I of *E. coli* strain K-12 substrain MG1655 (EO53_04950); Las-psy62 (CLIBASIA_04190); Las-gxpsy (WSI_04025) and Las-Ishi-1 (CGUJ_04190) strains (Supplementary Figure S1). The identity for the SPase I of the three Las strains is 100%, whereas the Las SPase I protein shares 34% identity and 52% similarity with that of *E. coli.*

**FIGURE 4:**
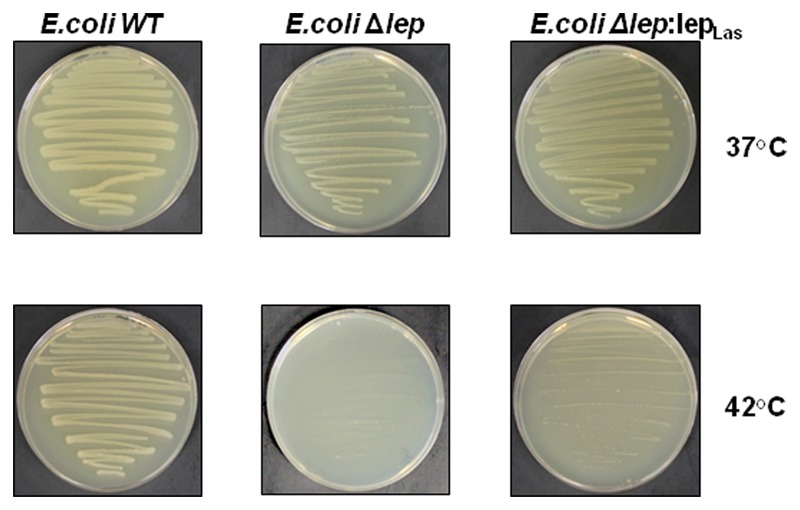
**Las *lepB* could partially complement the *lepB* mutant of *E.coli*.** *E.coli lepB* amber mutant grows normally at 37°C as wild type strain, but could not grow at 42°C. Complementation of *E.coli lepB* mutant with *lepB* homolog of Las partially restores the growth of the mutant strain at 42°C. WT: *E. coli* wild type (IT42: *lepB*), *E.coli*Δlep: *E. coli* (IT41: lep9) amber mutant and *E.coli*Δlep:lep_Las_: *E. coli* (IT41: lep9) amber mutant complimented with Las *lepB* gene.

We further tested whether Las *lepB* gene which encodes SPase I could complement the *E. coli* amber mutants of *lepB.* The *lepB* amber mutant of *E. coli* (Δ*lep*) displays temperature sensitivity, leading to conditional lethality at 42°C, but not at 37°C (Paetzel, 2014). At 37°C, the WT, Δ*lep* and the complimented Δ*lep:lep*_Las_ strains showed similar growth. At 42°C, the WT and Δ*lep:lep*_Las_ strains displayed growth, whereas the Δ*lep* strain was unable to grow (**Figure 4**). It is noteworthy that Δ*lep:lep*_Las_ grew slower than the wild type *E.coli* strain, which indicates that Las *lepB* could partially complement the *lepB* mutant of *E.coli.*

## Discussion

The signal peptide is an important protein-sorting signal that targets its passenger protein for transportation out of the cytoplasm in prokaryotes (Von Heijne, 1990). Many methods have been used for predicting signal peptides, including SignalP (Nielsen et al., 1997; Nielsen and Krogh, 1998; Bendtsen et al., 2004; Petersen et al., 2011), PrediSi (Hiller et al., 2004), SPEPlip (Fariselli et al., 2003), Signal-CF (Chou and Shen, 2007), Signal-3L (Shen and Chou, 2007), signal-BLAST (Frank and Sippl, 2008), Phobius (Käll et al., 2004), LipoP (Juncker et al., 2003) and Philius (Reynolds et al., 2008). All the prediction methods have limited ability to discriminate between signal peptides and N-terminal transmembrane helices. The common characteristic of signal peptides and N-terminal transmembrane helices is hydrophobic. Transmembrane helices usually have longer hydrophobic regions. Transmembrane helices do not have cleavage sites that are associated with signal peptides. However, the cleavage-site pattern alone is not sufficient to distinguish the two types of sequence. Consequently, each method has its pros and cons and both false positives and false negatives were reported for each prediction method (Heng Choo et al., 2009). Among them, SignalP, Phobius, and LipoP use distinct algorithms for prediction and complement each other. Specifically, Phobius combined transmembrane protein topology and signal peptide predictor, thus generating superior prediction in differentiating signal peptides from transmembrane helices. In addition, LipoP using hidden Markov model (HMM) can distinguish between lipoproteins (SPaseII-cleaved proteins), SPaseI-cleaved proteins, cytoplasmic proteins, and transmembrane proteins (Juncker et al., 2003). On the other hand, SignalP and most prediction programs are only trained on SPaseI-cleaved proteins (Nielsen et al., 1997; Nielsen and Krogh, 1998; Bendtsen et al., 2004; Petersen et al., 2011). Thus, we combined SignalP 3.0, SignalP 4.1, Phobius, and LipoP for prediction of SP-containing extracytoplasmic proteins in Liberibacters. In spite of the potential false positive and false negative predictions, it is believe the prediction is still useful since 87 to 96% accuracy have been reported for the various programs (Juncker et al., 2003; Heng Choo et al., 2009). The overlapping prediction results of SignalP 3.0 and 4.0, Phobius, and LipoP will likely to be accurate, but with false negative, whereas the overall predication results will likely remove false negative results, but with false positives. Thus, experimental confirmation is critical for the in silico predication of SP-containing extracytoplasmic proteins in Liberibacters.

Since Las has not been cultivated in media, we have used *E.coli* as a model to indirectly validate the predicated SP containing proteins with PhoA assay. Out of the 166 proteins predicted, 86 proteins were PhoA positive tested in *E. coli*, suggesting that they contain a SP in their sequences that can direct them to be translocated outside of the cytoplasm via the Sec-translocon. PhoA assay using *E.coli* as a model has been used to experimentally test SP-containing proteins in multiple bacteria including *Pseudomonas aeruginosa* (Lewenza et al., 2005), *Helicobacter pylori* (Bina et al., 1997), *Bacillus subtilis* (Payne and Jackson, 1991), *Actinobacillus actinomycetemcomitans* (Mintz and Fives-Taylor, 1999; Ward et al., 2001), *Mycobacterium tuberculosis* (Wiker et al., 2000), *Streptococcus pneumoniae* (Pearce et al., 1993), *Vibrio cholerae* (Taylor et al., 1989), *Staphylococcus aureus* (Williams et al., 2000) and *Rickettsia typhi* (Ammerman et al., 2008). A heterologous system could be used to test the secretion of SP-containing proteins by the Sec pathway is because that the SP and Sec apparatus are conserved. The Las Sec apparatus contains SecB, Ffh, SecE, SecD/F, YidC, YajC, SecY, and SecA which share 28–50% identity and 52–70% similarity with their counterparts in *E.coli*. The majority of signal peptides are cleaved by signal peptidase I which is encoded by *lepB* and shares 34% identity and 52% similarity with its counterpart in *E.coli* (Supplementary Figure S1). Type II signal peptides, which are associated with lipoproteins are cleaved by signal peptidase II. The signal peptidase of Las shares 37% identity and 57% similarity with that of *E.coli*. Las *lepB* could partially complement the *lepB* amber mutant of *E.coli* (**Figure 4**). The aforementioned evidence suggests that the PhoA assay using *E.coli* as a model will provide strong experimental support of confirmation of SP. Furthermore, among the 86 PhoA positive proteins, many are associated with the cell envelope including outer membrane proteins (e.g., OmpA/MotB, and Omp19), flagellar proteins, Type IV pilus proteins, proteases, dehydrogenases, hydrolase, monophosphatase, monooxygenase, ATPase, ABC transporters, periplasmic binding proteins, translocation protein, and nodulation related eﬄux protein (Supplementary Table S10), which further support the reliability of PhoA assay. Additionally, we need to point out that 29 predicated SP containing proteins were not determined in this study. Most of them are due to failure of amplification despite repeated attempts. Thus it is likely that more predicted SP containing proteins can be experimentally verified.

Remarkably, significantly high number of hypothetical proteins (47) were PhoA positive in *E. coli*, which is intriguing and certainly suggests the need for further investigation. Additionally, 36 SP containing proteins have been shown to be highly expressed in planta compared to in psyllids whereas eight are highly expressed in psyllids compared to in planta (Supplementary Table S11) (Yan et al., 2013), which suggest that those proteins might play critical roles for Las adapts to its living in the two hosts. In addition, CLIBASIA_04040 contains four known domains out of which two motifs (PF09487: HrpB2 and PF05758: Ycf1) have been shown to be involved in virulence in other plant pathogens, e.g., *P. syringae* and animal pathogens, e.g., *Yersinia*. How the SP-containing hypothetical extracytoplasmic proteins contribute to the virulence of Las remains to be explored.

As Las possesses a highly reduced genome size (1.23-Mb), presence of the Sec-translocon suggests the Sec-translocon and its substrates play important roles for Las and other *Liberibacters*. A total of 166 proteins were predicted to contain SP in Las-psy62 whereas 168 and 164 SP-containing extracytoplasmic proteins were predicated for Las-gxpsy and Las-ishi-1, respectively. The three Las strains from USA, China and Japan show high uniformity in their Sec dependent extracytoplasmic proteins with 151 overlapping in all three. This is consistent with the high ANI values (99.85–99.94%) of the three strains. The similarity in Sec dependent extracytoplasmic proteins and ANI indicate that the Las strains in US, China and Japan have not undergone extensive evolution changes despite the graphical separation. However, significant differences were observed between Las, Laf, and Lam even though they all cause HLB. Only 45 Sec dependent extracytoplasmic proteins showed homology between them. The significant difference in Sec dependent extracytoplasmic proteins in Las, Laf, and Lam might contribute to the virulence and/or adaption difference among the three Liberibacter species with Las being the most widely spread species.

## Conclusion

We predicted SP-containing extracytoplasmic proteins for Las, Lam, Laf, Lso, and Lcr. Eighty six Las proteins has been experimentally confirmed to be SP-containing extracytoplasmic proteins using PhoA assay with *E.coli* as a model. Our study has provided insight into the potential function of certain SP-containing hypothetical proteins of Las. Our data also showed that Las *lepB* gene can partially complement the *E.coli lepB* amber mutant. Due to the importance of Sec-translocon and its substrate, suppression of the Sec secretion system by developing antimicrobials targeting suitable targets, e.g., SecA, has the potential to inhibit HLB progression (Akula et al., 2011).

## Author Contributions

NW, SP, JX, and YZ initiated the project and designed experiments. SP, JX, YZ, and NW performed all experiments and data analysis. NW, SP, JX, and YZ wrote the manuscript. NW supervised the project.

## Conflict of Interest Statement

The authors declare that the research was conducted in the absence of any commercial or financial relationships that could be construed as a potential conflict of interest.

## Footnotes

1 http://genomes.urv.es/UPGMA/

2 http://www.endmemo.com/bio/tm.php
