# Supplementary material for: SEC-Translocon Dependent Extracytoplasmic Proteins of Candidatus Liberibacter asiaticus
Source: Front Microbiol. 2016 Dec 20;7:1989. doi: 10.3389/fmicb.2016.01989 (PMC5167687; doi:10.3389/fmicb.2016.01989)
Supplement: Supplementary file 2 [file Image_1.pdf]

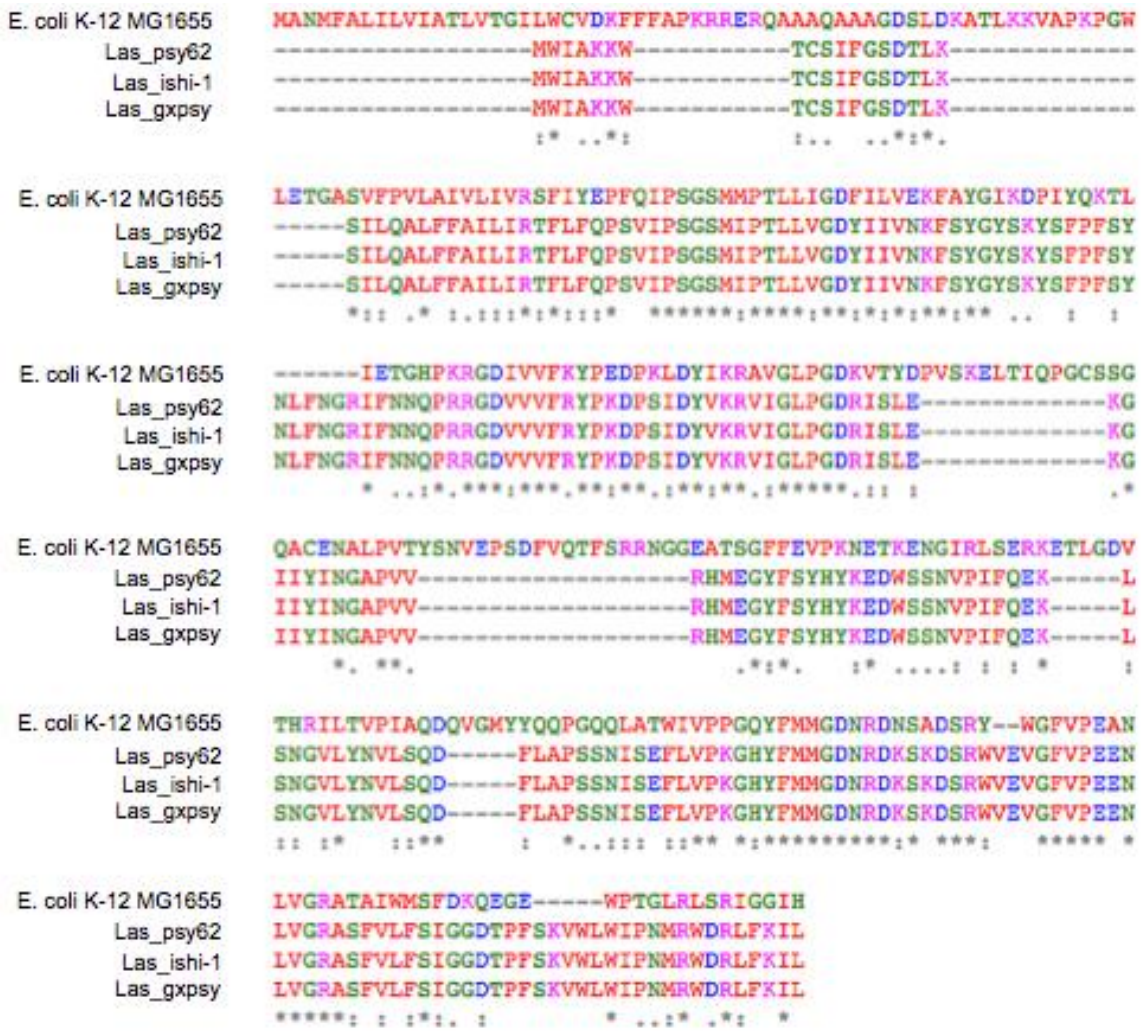

Supplementary Figure 1. Multiple sequence alignment of signal peptidase I of Las_psy62, Las_gxpsy, Las_ishi-1 strains and *E. coli* strain K-12 sub. Str. MG1655. The MUSCLE version 3.8 alignment online tool at default settings was used. Conserved residues are denoted by “*”, residues of similar polarity are denoted by “.”, residues of different polarity are denoted by “:” and Gaps are denoted by “-”.
